# “Nano-Ginseng” for Enhanced Cytotoxicity AGAINST Cancer Cells

**DOI:** 10.3390/ijms19020627

**Published:** 2018-02-23

**Authors:** Lin Dai, Weiyan Zhu, Chuanling Si, Jiandu Lei

**Affiliations:** 1 Tianjin Key Laboratory of Pulp and Paper, College of Papermaking Science and Technology, Tianjin University of Science and Technology, Tianjin 300457, China; ywzhu77@163.com (W.Z.); sichli@tust.edu.cn (C.S.); 2 Beijing Key Laboratory of Lignocellulosic Chemistry, College of Materials Science and Technology, Beijing Forestry University, Beijing 100083, China

**Keywords:** nanoparticle, drug delivery, self-assemble, anticancer, green process

## Abstract

*Panax ginseng* has high medicinal and health values. However, the various and complex components of ginseng may interact with each other, thus reducing and even reversing therapeutic effects. In this study, we designed and fabricated a novel “nano-ginseng” with definite ingredients, ginsenoside Rb1/protopanaxadiol nanoparticles (Rb1/PPD NPs), completely based on the protopanaxadiol-type extracts. The optimized nano-formulations demonstrated an appropriate size (~110 nm), high drug loading efficiency (~96.8%) and capacity (~27.9 wt %), long half-time in systemic circulation (nine-fold longer than free PPD), better antitumor effects in vitro and in vivo, higher accumulation at the tumor site and reduced damage to normal tissues. Importantly, this process of “nano-ginseng” production is a simple, scalable, green economy process.

## 1. Introduction

For thousands of years in traditional Chinese medicine, *Panax ginseng* has been regarded as having “amazing medicinal values”. Virtually the active agents are the extracts of ginseng, including polysaccharides, flavonoids, volatile oils, and ginsenosides [1]. Protopanaxadiols (PPD-type), a kind of ginsenoside with dammarane structures, are the most important active ingredients obtained from ginseng species. Based on molecular structure, most of the ginsenosides belong to the PPD-type group. In the PPD-type group, the β-hydroxy at C-3 and C-20 of the aglycone are connected with sugar residues, such as ginsenosides Ra1, Ra2, Rg3, Rh2, and Rb1 [2,3]. When ginseng was used directly for pharmaceutical purposes, some unexpected effects may have been due to the interaction of the various and complex components of ginseng. In some recent years, many active and inactive PPD-type ginsenosides have been separated and widely investigated. Among these chemical entities, PPD, without any sugar residues, showed the greatest efficacy against cancer cells [4,5]. Although promising, the application of PPD is still limited by its low molecular weight, short half-time, and strong hydrophobicity [6]. Due to these limitations, it is necessary to develop PPD carriers.

Nano-sized particulate platforms or nanoparticles (NPs) have proven to be of enormous potential in biological studies, diagnosis and in the treatment of cancer [7,8,9]. Depending on the particle size and surface properties, engineered nanoparticles may demonstrate several unique advantages, including high surface-to-volume ratio and high bioavailability. Core-shell structure nanoparticle is one kind of nano-drug delivery system, which originates from the spontaneous self-assembly of amphiphilic molecules in an aqueous environment [10,11]. This kind of nanoparticle typically consists of at least two components, the pharmaceutically active ingredient, and the excipient. Traditional drug excipients were developed by synthetic or semi-synthetic inert polymers which are minimally absorbed by the organism. Exploration and application of green materials for drug delivery not only can improve drug safety but can also meet environmental and economic sustainability objectives.

Ginsenoside Rb1, a kind of PPD-type ginsenoside with four sugar molecules, was reported to be amphipathic, anti-angiogenic and have weak anti-proliferative effects [12,13]. Rb1 would be a potential adjuvant to improve the solubility and performance of anticancer drugs. Moreover, the Rb1 and PPD molecular structures are of the same part of dammarane-type, which can easily form the self-assembled and PPD-loaded nanoparticles. In this study, “nano-ginseng”, ginsenoside Rb1/protopanaxadiol nanoparticles (Rb1/PPD NPs), were designed and fabricated. The physicochemical properties and anticancer efficiency were also investigated systematically.

## 2. Results

### 2.1. Formulation of Rb1/PPD Nanoparticles (NPs)

**Figure 1:**
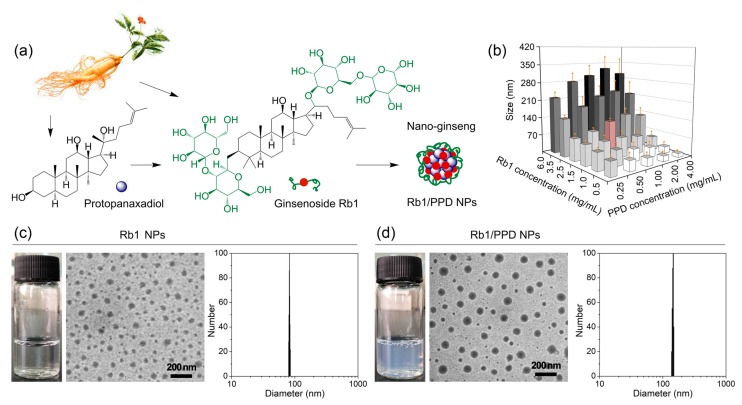
(**a**) Illustration; (**b**) Formation of ginsenoside Rb1/protopanaxadiol nanoparticles (Rb1/PPD NPs) (*n* = 3); and (**c**,**d**) optical, TEM images, and drug loading capacity (DLC) results.

The nano-ginseng delivery system (ginsenoside Rb1/protopanaxadiol nanoparticles, Rb1/PPD NPs) was fabricated from two ginseng (20S)-protopanaxadiol type compounds, Rb1 and PPD. The PPD and the hydrophobic component of Rb1, with the same structures, can aggregate and self-assemble to form inner hydrophobic cores. The sugar residues of Rb1 molecules form the shell outside of the NPs which enhances the stability and water dispersibility of this nano-system (Figure 1a). The desired size of Rb1/PPD NPs was elucidated by optimizing the concentration of Rb1 and PPD from 0.5 to 6 mg/mL and 0.25 to 4 mg/mL, respectively (Figure 1b). The sizes of the nanoparticles were increased with further additions of PPD. After a certain point, the size of the NPs would decrease with further addition of PPD. A nano-delivery system with a particle size of approximately 120 nm could exhibit improved performance of passive targeting via the enhanced permeability and retention (EPR) effect in vivo [14,15]. Moreover, 1 mg/mL PPD and 2.5 mg/mL Rb1 were selected for optimal conditions of Rb1/PPD NPs preparation. The Rb1/PPD NPs with a 96.8% drug loading efficiency (DLE) and 27.9 wt % drug loading capacity (DLC) were chosen for further anticancer tests in vitro and in vivo. As seen in Figure 1c,d, perfect sphere and good uniformity were observed for blank Rb1 NPs and Rb1/PPD NPs. Moreover, the PPD-loaded NPs were larger in size compared to blank ones.

### 2.2. Drug Stability In Vitro

**Figure 2:**
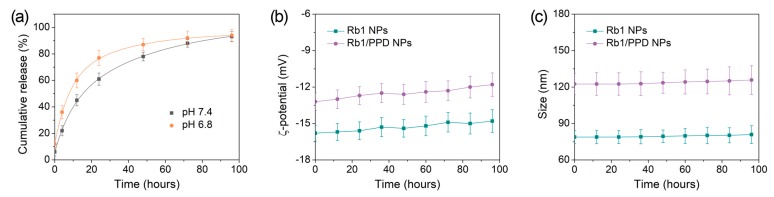
(**a**) Release kinetics in pH 7.4 and 6.8 at 37 °C. The stability of (**b**) ζ-potential and (**c**) Size in pH 7.4 of the samples at 4 °C (*n* = 3).

The PPD release behaviors from the Rb1 nano-delivery system were detected in phosphate buffered saline (PBS) solutions (at pH 7.4 and 6.8), which simulated the blood and the intracellular environment. The results of PPD payload and in vitro release profile showed relatively high encapsulation efficiency, drug loading capacity, and slow release kinetics in Rb1 NPs (Figure 2a, Table 1 and Table 2), which likely leads to a strong interaction between PPD and Rb1. The hydrophobic part of Rb1 has the same dammarane-type structures as PPD. In addition to hydrophobic interactions with PPD, the hydrogen bonding, and π–π stacking also enhances the interaction of Rb1 and PPD [16]. In Figure 2a, more than 20% of PPD was released in the initial stage. This phenomenon of sudden release may be due to PPD molecules loaded on the interface of the core-shell or on the shell of NPs, the concentration gradient, and osmotic pressure. The release kinetics were further evaluated using different kinetic models [17,18]. Table 2 shows that the Higuchi model best fit the experimental data, which suggests that the release of PPD from Rb1/PPD NPs was a diffusion process based on Fick’s law, square root time dependent.

The ζ-potential is another critical factor to evaluate the stability of NP dispersion. Due to the attachment of sugar residues on C-3 and C-20 of the aglycone (PPD), Rb1 NPs and Rb1/PPD NPs carried negative charges on their surfaces which had positive effects on the interaction with cancer cells. It is generally regarded that the internally negatively charged nanoparticles can be internalized by first clustering, then through nonspecific binding on cationic sites of the plasma membrane, followed by their subsequent endocytosis [19]. In addition, as shown in Figure 2b,c, the ζ-potential and average sizes of the nanoparticles had changed little; Rb1 NPs and Rb1/PPD NPs had good re-dispersion stability during the investigation period.

### 2.3. Hemolysis Study

**Figure 3:**
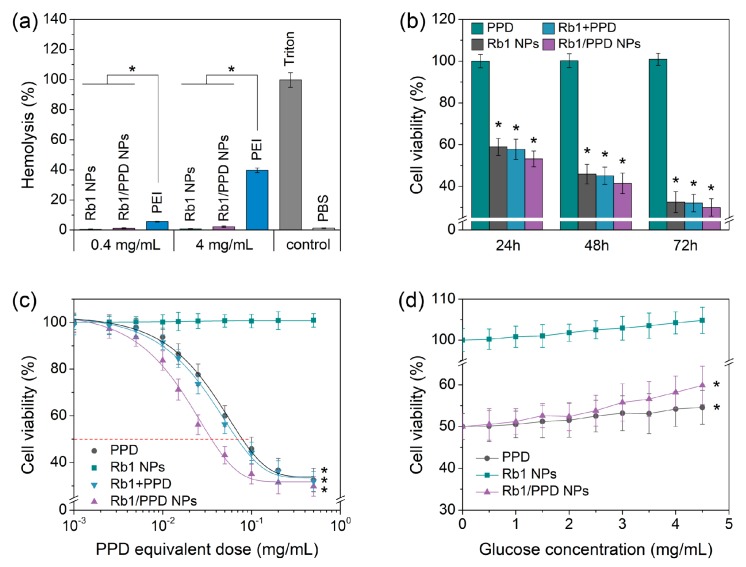
(**a**) Hemolysis (*n* = 3), cell viability with (**b**) different times, (**c**) different concentrations, and (**d**) glucose competitions with Rb1 NPs and Rb1/PPD NPs (*n* = 3). * indicates a *p*-value of less than 0.05.

Intravenous (IV) medications should be assessed to ensure minimal detrimental interactions with red blood cells (RBCs). Hemolysis was evaluated by incubating erythrocytes with different samples, in 0.4 and 4 mg/mL concentrations, for 1 h at 37 °C, followed by the measurement of the amount of hemoglobin released into the supernatant at 541 nm. PBS and Triton X-100 were used as negative (0% release of hemoglobin) and positive controls (100% release of hemoglobin), respectively. Also, polyethyleneimine (PEI) with a molar mass of 25 kD (PEI_25K_), a cationic polymer known to have significant hemolytic properties, was used as the reference sample. Figure 3a highlights the extremely low hemoglobin releases of Rb1 NPs and Rb1/PPD NPs at different concentrations. The results suggested that this nano-ginseng system, Rb1/PPD NPs, is safe and without side effects.

### 2.4. In Vitro Cytotoxicity

The ability to kill cancer cells is an important indicator that can be used to evaluate the effectiveness of the drug and delivery system. Previous studies have shown that PPD (with no sugar residues) inhibits different types of cancer cells [5,20]. In this work, the cytotoxicity of free PPD, Rb1 NPs, and Rb1/PPD NPs were evaluated using the Cell Counting Kit-8 (CCK-8) assay with murine Lewis lung carcinoma (LLC) cells. Of note, Rb1 NPs + PPD, stated in this article refers to the addition of Rb1 NPs and PPD directly (the concentration of Rb1 is 10 μg/mL, and PPD concentrations in Rb1 NPs + PPD and Rb1/PPD NP groups were equal to native PPD). In Figure 3b, LLC cell death was dependent upon the incubation time of free PPD and PPD formulations, which indicated superior anticancer activity of Rb1/PPD NPs compared to other groups.

To explore the efficacy of Rb1/PPD NPs, the concentrations used to kill half of the cancer cells (IC_50_) were obtained from cell viability curves. As can be seen from Figure 3c, the IC_50_ value of Rb1/PPD NPs (35.32 ± 4.30 μg/mL) was significantly less than that of free PPD (78.81 ± 5.36 μg/mL), which may be due to the internalization and slow release of the nano-formulation. To further clarify, the direct addition of Rb1 and free drug on cell viability was compared with the PPD and Rb1/PPD NP treatment groups. It should be noted that the concentrations of different formulations refer to PPD equivalents. And the Rb1 concentrations in Rb1 + PPD group were equaled with that in Rb1/PPD NPs. For example, a 10 μg/mL dose of Rb1 + PPD contains 2.79 μg/mL of PPD and 7.21 μg/mL of Rb1, assuming that the loading of PPD in Rb1/PPD NPs is 27.9%. Clearly, the synergistic effect of combined Rb1 and PPD can improve the inhibition rate of LLC cells compared to free PPD. In summary, the combination of nano and synergistic effects could explain the significantly greater anticancer activity of Rb1/PPD NPs.

### 2.5. Glucose Competition

Previous studies have suggested that ginsenosides with four or more sugar molecules, such as Rb1, have no significant anti-proliferative effects, in agreement with present findings. Glucose dependence in metabolic processes may be the most relevant and dramatic difference between cancer and normal tissues. Tumors seek glucose avidly, as they rely on glucose as an important source of carbon and energy [21]. Precisely using this mechanism, glucose, and its analogs could serve as target molecules and be applied to drug delivery. In order to confirm the role of the sugar side chains in the cellular uptake of nanoparticles, the LLC cells were treated with the nanoparticles at IC_50_ concentration, with culture media also containing increasing concentrations of glucose. From the results shown in Figure 3d, the cell viability of the Rb1/PPD NPs group increased with increasing glucose concentration. However, a clear change as a function of glucose concentration was not observed for free PPD. For instance, the cell viability of Rb1/PPD NPs against LLC cells was 50% without glucose and 51.2% at glucose concentrations of 1.0 mg/mL, but when the glucose concentration rose to 4.5 mg/mL the LLC cells viability was reaching nearly 60%. This phenomenon may be due to the competition between glucose molecules and sugar residues on the surface of Rb1/PPD NPs.

### 2.6. In Vivo Biodistribution and Pharmacokinetics

**Figure 4:**
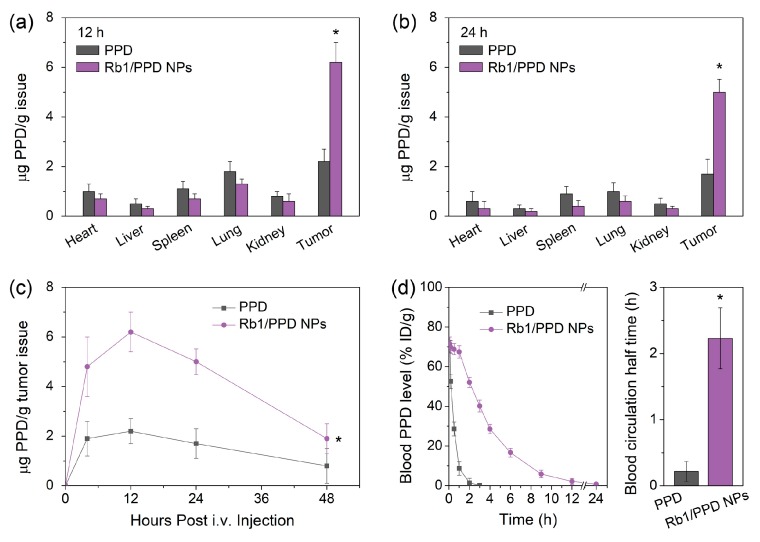
Biodistribution with the PPD and Rb1/PPD NPs for (**a**) 12 h and (**b**) 24 h (*n* = 3); (**c**) PPD content in tumor issues (*n* = 3); and (**d**) Blood concentration level of PPD and Rb1/PPD NPs (*n*= 3). * indicates a *p*-value of less than 0.05.

Nano-delivery is an effective approach to optimize the biodistribution and improve the bioavailability of chemotherapeutic drugs. In this work, in vivo biodistributions of PPD were evaluated on LLC-tumor bearing C57BL/6 mice. When the tumor volume reached approximately 300 mm^3^, a single IV dose of free PPD (10 mg/kg) and Rb1/PPD NPs (10 mg respiratory syncytial virus; RSV-equivalent/kg) was administered to all mice. After 12 and 24 h, respectively, the PPD contents in different organs were analyzed quantitatively. Compared to free PPD, Rb1/PPD NPs showed a high level of PPD accumulation in the tumor tissues and low levels of PPD accumulation in the liver, spleen, lung, and kidney. In Rb1/PPD NPs group, it should be noted that the at 24 h post-medication administration, PPD content in the tumor still remained at a relatively high level. The area under the concentration–time curve (AUC) of Rb1/PPD NPs increased six times compared with free PPD (Figure 4c), which was evidently attributed to the EPR and some degree of a glucose-targeted effect. These results show that Rb1/PPD NPs would achieve greater accumulation in the tumor site and result in less damage to some normal tissues.

An ideal drug delivery system also requires a long blood circulation time. PPD has a low molecular weight which led to very rapid disappearance in vivo within 3 h after IV injection (Figure 4d). Rb1/PPD NPs, by contrast, show an obvious prolonged clearance with the PPD levels of approximately 18% of the injected dose per gram (% ID/g), at 6-h post-IV injection, and dramatically extended (nine-fold longer) the blood circulation half-time of PPD from 0.4 to 3.6 h.

### 2.7. In Vivo Anticancer Activities

**Figure 5:**
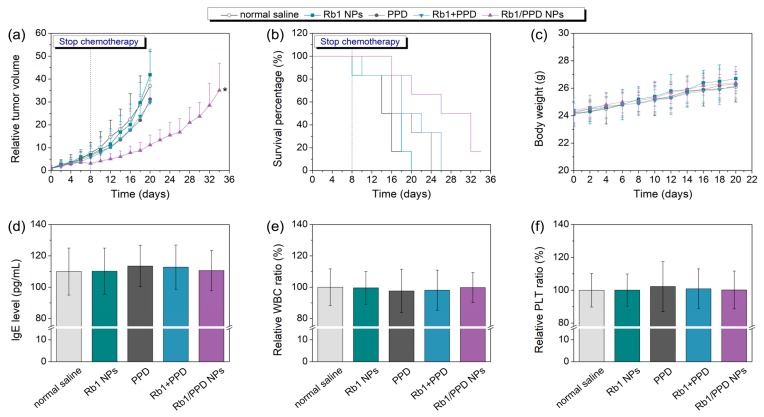
(**a**) Tumor inhibition, (**b**) survival rates, (**c**) body weight of tumor-bearing mice (*n* = 6), and (**d**) Immunoglobulin E (IgE) level, (**e**) relative ratios of white blood cells (WBC), and (**f**) platelets (PLT), with different samples (*n* = 6). During the development process of biomedical materials, hypersensitivity, bleeding, and other adverse reactions should be avoided to the greatest extent. Here, the immunoglobulin E (IgE) antibody levels, white blood cell (WBC) number and platelets (PLT) of different treatments were detected. As expected, the administration of Rb1 NPs and Rb1/PPD NPs maintained normal IgE levels. There were no significant changes to the WBC and PLT ratios, highlighting the security of this nano-delivery system. * indicates a *p*-value of less than 0.05.

After in vitro tests, the efficacy of Rb1/PPD NPs was further evaluated via animal models. Firstly, LLC subcutaneous xenograft models were fabricated by injecting 3 × 10^6^ LLC cells into C57BL/6 mice (female, 6–7 weeks). When the average tumor volume reached about 100 mm^3^, free PPD (20 mg/kg), Rb1 NPs, Rb1/PPD NPs (20 mg PPD-equivalent/kg) were injected into the mice, respectively. Free PPD demonstrated good efficacy against cancer cells in vitro, due to the poor water-solubility and short half-life, in vivo performance of PPD was barely satisfactory. As shown in Figure 5a and Table 3, the Rb1/PPD NPs groups showed better therapeutic effect (71.3% of tumor growth inhibition at day 20) and higher survival rate (33.3% survival at day 30). Rb1/PPD NPs had a satisfactory curative performance, which may be advantageous for the following reasons: (i) appropriate particle size (approximately 100 nm) to better achieve the EPR effect; (ii) a nano-delivery system may improve PDD dispersibility; and (iii) the sugar-shell of Rb1/PPD NPs could increase the accumulation in the tumor site and decrease the damage to surrounding normal tissues.

## 3. Materials and Methods

### 3.1. Materials

Ginsenoside Rb1 (Rb1) and protopanaxadiol (PPD) were bought from Chengdu Preferred Biotechnology Co., Ltd. (Chengdu, China). Other reagents were offered from Sinopharm Chemical Reagent Co., Ltd. (Tianjin, China). Murine Lewis lung carcinoma (LLC) cells were supplied by the Peking University Health Science Center, cultured by Dulbecco’s Modified Eagle’s Medium (DMEM) with 10% fetal bovine serum (FBS), 1% streptomycin-penicillin and maintained at 37 °C with 5% CO_2_/95% air humidified atmosphere. CCK-8 was purchased from the Dojindo Laboratories (Shanghai, China).

### 3.2. Animals and Ethics

C57BL/6 mice (female, 6–7 weeks of age) were supplied by HFK Bioscience Co., Ltd. (Beijing, China). The mice were housed in the animal laboratory of the Institute of Process Engineering (CAS, Beijing, China) in a climate-controlled environment (22 ± 2 °C, 55 ± 5% relative humidity level, a 12-h light/dark cycle). All animal experiments were in line with National Institutes of Health regulations and were approved by the Beijing Experimental Animal Ethics Committee (the National Institutes of Health publication No. 85-23, revised 18 March 1985).

### 3.3. Preparation of Rb1/PPD Nanoparticles (NPs)

A precipitation method was used to prepare Rb1 NPs and PPD-loaded nanoparticles. Briefly, PPD and Rb1 were dissolved in dimethyl sulfoxide (DMSO; 0.5 mL) at different concentrations, followed by injection into the water (3.5 mL) under magnetic stirring for 10 min. The resulting Rb1/PPD NP solutions were dialyzed in normal saline using a cartridge with a 1 kDa molecular weight cut-off for 12-h with two exchanges of dialysate. The particles sizes were detected by dynamic light scattering with a Malvern Zetasizer Nano-ZS particle analyzer. The percent yield of the nanoparticles was calculated as follows:

(1) $$\mathrm{Yield}\left(\%\right)=\frac{\mathrm{weight}\;\mathrm{of}\;\mathrm{nanoparticles}}{\mathrm{weight}\;\mathrm{of}\;\mathrm{Rb}1\;\mathrm{and}\;\mathrm{PPD}}\times 100\%$$

### 3.4. Characterization of Nanoparticles

One drop of diluted nanoparticles solution was transferred onto the surface of Formvar-coated copper grids (Beijing XXBR Technology, Beijing, China). After being naturally air dried, the observation was processed on a JEM-100CXa transmission electron microscope at 100 kV. The particle size and ζ-potential were detected by a Malvern Nano-ZS Zeta Sizer.

### 3.5. Drug Loading and Release

The releases of PPD from the Rb1/PPD NPs was analyzed by a dialysis method. 5 mL PPD-loaded nanoparticle solution (5 mg/mL Rb1/PPD NPs) was transferred into a cartridge with a 1 kDa molecular weight cut-off, then immersed into PBS buffer (200 mL, pH 7.4 or 6.8) with gentle shaking at 37 °C. The PPD concentrations at different times in PBS buffer were detected by high-performance liquid chromatography (HPLC), which employed a VYDAC 214TP54 (C18, 5 μm, 4.6 × 250 mm) with a 1 mL/min flow rate of 95% methanol solution and was analyzed at 203 nm using a UV detector. Drug loading capacity (DLC) and efficiency (DLE) were calculated using the following equations:

(2) $$\mathrm{DLC}\left(\mathrm{wt}\%\right)=\frac{\mathrm{weight}\;\mathrm{of}\;\mathrm{PPD}\;\mathrm{in}\;\mathrm{nanoparticles}}{\mathrm{weight}\;\mathrm{of}\;\mathrm{PPD}-\mathrm{loading}\;\mathrm{nanoparticles}}\times 100\%$$

(3) $$\mathrm{DLE}\left(\%\right)=\frac{\mathrm{weight}\;\mathrm{of}\;\mathrm{PPD}\;\mathrm{in}\;\mathrm{nanoparticles}}{\mathrm{weight}\;\mathrm{of}\;\mathrm{PPD}\;\mathrm{added}\;\mathrm{initially}}\times 100\%$$

In addition, four different kinetic models: first order kinetic, Hixson–Crowell model, Higuchi model and Korsmeyer–Peppas model were used to explore the behavior of PPD release from Rb1/PPD NPs.

(4) $$\mathrm{First}\;\mathrm{order}\;\mathrm{kinetic}:\log Q_{t}=\log Q_{0}+\frac{kt}{2.303}$$

(5) $$\mathrm{Hixson}–\mathrm{Crowell}\;\mathrm{model}:Q_{0}^{1/3}-Q_{t}^{1/3}=kt$$

(6) $$\mathrm{Higuchi}\;\mathrm{model}:Q_{t}=kt^{0.5}$$

(7) $$\mathrm{Korsmeyer}–\mathrm{Peppas}\;\mathrm{model}:\frac{Q_{t}}{Q_{\infty }}=kt^{n}$$

where *Q_t_* is the amount of drug released in time *t*, *Q*_0_ and *Q*_∞_ is the initial and final amount of PPD in the solution, respectively. *k* is the constant of different models, and *n* is the release exponent of the Korsmeyer–Peppas model.

### 3.6. Hemolysis Assay

The fresh whole blood samples (10 mL) were collected for detecting hemolytic activity. The red blood cells (RBCs) were separated (1500 rpm, 10 min at 4 °C), washed, and suspended in ice-cold Dulbecco’s phosphate-buffered saline (DPBS) to obtain a 5 × 10^8^ cells/mL suspension. A 4 mL Rb1 NPs, Rb1/PPD NPs, or PEI_25K_ solution was mixed and incubated with the same volume of water, respectively, at 37 °C for 1-h. The positive and negative controls were 1% Triton X-100 in DPBS, and pure DPBS, respectively. After centrifuging (1500 rpm, 10 min at 4 °C), the release of hemoglobin was detected at 541 nm using the Tecan Infinite M200 microplate spectrophotometer from three independent experiments and calculated with the following equation:

(8) $$\mathrm{Hemoglobin}\;\mathrm{release}\left(\%\right)=\frac{\mathrm{OD}\;\mathrm{value}\;\mathrm{of}\;\mathrm{the}\;\mathrm{sample}-\mathrm{OD}\;\mathrm{value}\;\mathrm{of}\;\mathrm{the}\;\mathrm{negative}\;\mathrm{control}}{\mathrm{OD}\;\mathrm{value}\;\mathrm{of}\;\mathrm{the}\;\mathrm{positive}\;\mathrm{control}-\mathrm{OD}\;\mathrm{value}\;\mathrm{of}\;\mathrm{the}\;\mathrm{negative}\;\mathrm{control}}\times 100\%$$

### 3.7. In Vitro Cell Cytotoxicity

Cytotoxicity was evaluated using LLC cells for different times. Cells were seeded in 96-well tissue culture plates in 180 μL culture medium at the concentration of 3 × 10^3^ cells/well and incubated for 24 h at 37 °C. Serial dilutions of 20 μL samples (PPD, Rb1 NPs, Rb1 NPs + PPD, and Rb1/PPD NPs) were added and continued to incubate for 24, 48, and 72 h. Then, the cytotoxicity was measured by the CCK-8 assay. Cell viabilities of different groups were calculated by detecting the absorbance at 450 nm. The IC_50_ (half-inhibitory concentration) was calculated using the Boltzmann sigmoidal function [22].

### 3.8. Pharmacokinetic Experiments

Female C57BL/6 mice were treated by free PPD and Rb1/PPD NPs via tail vein injection. During the administration, the blood samples (~200 μL) were collected at timed intervals and centrifuged immediately (4 °C, 3000 rpm, 10 min). Next, the plasma sample was mixed, sonicated, and centrifuged with an equal volume of methanol. The clear supernatant was reconstituted by 100 μL methanol for HPLC measurement, as mentioned above [23].

### 3.9. In Vivo Biodistribution

In Vivo biodistributions of PPD were evaluated on LLC-tumor bearing C57BL/6 mice (female, 6−7 weeks). The mice were randomly divided into five groups (*n* = 6), each received a single IV dose of free PPD and Rb1/PPD NPs when the tumor volume reached approximately 300 mm^3^. RSV dose in all injected mice was 10 mg/kg of body weight. The mice were sacrificed 12-h and 24-h post-injection, respectively. Heart, liver, spleen, lungs, kidneys, and tumor tissues were removed immediately. The tissue samples (approximately 100 mg of tissue) were transferred into vials, combined with 1 mL of methanol, and stainless-steel beads, and were homogenized at 3600 rpm for 5 min at 4 °C. To determine the PPD content in tissue samples, the organic layer was collected by centrifuging at 15,000 rpm for 5 min. After evaporating, PPD was re-dissolved in methanol (200 mL). Similar processes were performed on blank tissue samples. All the obtained PPD solutions were analyzed via HPLC as described above. PPD contents were plotted as the RSV level in tissues (μg PPD/g) against time after IV injection.

### 3.10. In Vivo Efficacy Studies

C57BL/6 mice (female, 6−7 weeks) were induced by injection of 3 × 10^6^ LLC cells to prepare LLC subcutaneous xenograft models. When the average tumor volume reached about 100 mm^3^, mice were treated by IV injection every other day with normal saline (control), Rb1 NPs, free PPD (10 mg/kg, DMSO content <0.5%), Rb1 + PPD, and Rb1/PPD NPs (10 mg PPD-equivalent/kg), respectively (with each injection administered for a total of 5 times, *n* = 6 per each group). The antitumor efficiencies were evaluated by measuring the tumor volume (TV), relative volume (RTV), and growth inhibition (TGI %) of the tumor with the use of the following equation:

(9) $$\mathrm{TV}=\frac{1}{2}\times \mathrm{longset}\;\mathrm{tumor}\;\mathrm{diameter}\times {\mathrm{longset}\;\mathrm{tumor}\;\mathrm{diameter}}^{2}$$

(10) $$\mathrm{RTV}=\frac{\mathrm{TV}\;\mathrm{of}\;\mathrm{the}\;\mathrm{treatment}\;\mathrm{group}}{\mathrm{TV}\;\mathrm{of}\;\mathrm{initial}\;\mathrm{treatment}}$$

(11) $$\mathrm{TGI}=\left(1-\frac{\mathrm{TV}\;\mathrm{of}\;\mathrm{the}\;\mathrm{treatment}\;\mathrm{group}}{\mathrm{TV}\;\mathrm{of}\;\mathrm{the}\;\mathrm{control}\;\mathrm{group}}\right)\times 100\%$$

The body weights and survival rates of the mice were monitored every day. All experimental mice would be sacrificed after more than 6 weeks or a tumor volume larger than 5000 mm^3^. After final administration, the blood samples (200 μL) were collected from each mouse to further evaluate the changes in WBC and PLT of different samples. In addition, IgE levels of different treatments were also tested using enzyme-linked immunosorbent assay (ELISA) methods [24,25]. Briefly, 96-well plates for ELISA were coated with anti-mouse IgE antibody by incubation at 4 °C for 12 h and then washed by with 2% (*w*/*v*) Bovine serum albumin (BSA) dissolved in PBS solution (containing 2% BSA and 0.05% Tween 20). Next, the serum samples (180 μL) were added in the wells and incubated for 1 h at room temperature. Then biotin-conjugated rat anti-mouse IgE antibody and streptavidin-peroxidase were added, respectively. The plates were developed using a substrate solution containing 0.04% *O*-phenylenediamine dissolved in phosphate-citrate buffer (pH 5.0). The reactions were terminated by the addition of sulfuric acid. The plates were read in a microplate reader at 490 nm and calculated by comparison with the mouse IgE standard.

### 3.11. Statistical Analysis

At minimum, each experiment was completed in triplicate. All data is presented as the mean ± standard deviation (SD). The *t*-test and one-way analysis of variance (ANOVA) were performed with statistical significance set at a *p*-value of less than 0.05. 

## 4. Conclusions

The formulations of ginseng with definite ingredients would promote the biomedical application of this traditional and venerable plant. In this study, we have designed and fabricated a novel nano-formulation completely based on the PPD-type extracts from ginseng (PPD and Rb1) which showed the advantages of controlling particle size and distribution, high efficiency, enhanced anticancer efficiency, and excellent biocompatibility. Above all, this green economy process is easy and scalable.

## Figures and Tables

**Table 1:** Characteristics and drug loading properties of the samples (*n* = 3).

Compound	Size (nm)	PDI	ζ-Potential (mV)	DLE (%)	DLC (wt %)	Yield (%)
Rb1 NPs	78.9 ± 8.2	0.061 ± 0.002	−15.8 ± 0.9	-	-	
Rb1/PPD NPs	122.5 ± 10.8	0.089 ± 0.005	−13.2 ± 0.6	96.8 ± 1.2	27.9 ± 1.4	89.6 ± 1.8

**Table 2:** Characteristics and drug loading properties of the samples (*n* = 3).

pH	First Order		Hixson–Crowell		Higuchi		Korsmeyer–Peppas		
	*k*	*r*^2^	*k*	*r*^2^	*k*	*r*^2^	*k*	*n*	*r*^2^
7.4	0.038	0.744	0.064	0.675	10.476	0.991	0.100	0.527	0.976
6.8	0.029	0.715	0.065	0.618	11.246	0.957	0.188	0.394	0.949

**Table 3:** Anticancer efficacy in vitro and in vivo of different groups (*n* = 3).

Compound	IC_50_ (μg/mL)	Mean TV ± SD (mm^3^) ^1^	RTV ^1^	TGI (%) ^1^	Cures (%) ^1^
normal saline	-	4225 ± 1748	37.0 ± 15.2	0	0
Rb1 NPs	-	4273 ± 1142	41.9 ± 11.2	-	0
PPD	78.81 ± 5.36	3526 ± 1220	31.2 ± 10.8	16.6	33.3
Rb1 + PPD	69.16 ± 5.08	3338 ± 1086	29.8 ± 9.7	21.0	50.0
Rb1/PPD NPs	35.32 ± 4.30	1322 ± 507	11.2 ± 4.3	68.7	66.7

^1^ Mean tumor volume (TV), relative volume (RTV), growth inhibition (TGI), and cures data on 20 days.
